# Physical Activity and Sedentary Behavior in University Students–The Role of Gender, Age, Field of Study, Targeted Degree, and Study Semester

**DOI:** 10.3389/fpubh.2022.821703

**Published:** 2022-06-16

**Authors:** Dennis Edelmann, Daniel Pfirrmann, Sebastian Heller, Pavel Dietz, Jennifer L. Reichel, Antonia M. Werner, Markus Schäfer, Ana N. Tibubos, Nicole Deci, Stephan Letzel, Perikles Simon, Kristin Kalo

**Affiliations:** ^1^Department of Sports Medicine, Disease Prevention and Rehabilitation, Johannes Gutenberg University Mainz, Mainz, Germany; ^2^Institute of Occupational, Social and Environmental Medicine, University Medical Centre of the University of Mainz, Mainz, Germany; ^3^Department of Psychosomatic Medicine and Psychotherapy, University Medical Centre, Johannes Gutenberg University Mainz, Mainz, Germany; ^4^Department of Communication, Johannes Gutenberg University Mainz, Mainz, Germany; ^5^Diagnostics in Healthcare and E-Health, University of Trier, Trier, Germany; ^6^Department of Work and Organizational Psychology, Faculty of Psychology, Medical School Hamburg, Hamburg, Germany

**Keywords:** physical activity, sitting time, student health, modifiable health influencing factors, sedentary behavior, university students

## Abstract

**Background:**

Physical inactivity and sedentary behavior are modifiable risk factors for an unhealthy lifestyle in university students. The aim of this study was to identify subgroups among German university students with an increased risk for unhealthy behavior. For this purpose, differences in physical activity and sedentary behavior with respect to sociodemographic and study related factors were examined.

**Methods:**

A total of 4,351 students participated in an online survey. The amount of physical activity (metabolic equivalent of task-min/week) and the sitting time (h/day) were assessed using the German short form of the International Physical Activity Questionnaire. Differences in gender and age as well as field of study, targeted degree and study semester were analyzed using a single factorial ANOVA with Tukey correction or a Welch-ANOVA with Games-Howell correction.

**Results:**

For physical activity, significant differences were found for gender (*F*_(2, 80.46)_ = 17.79, *p* < 0.001, ηp2 = 0.009), for field of study (*F*_(5, 1738.09)_ = 7.41, *p* < 0.001, ηp2 = 0.01), and for study semester (*F*_(1, 948.12)_ = 5.53, *p* < 0.05, ηp2 =0.001), but not for age and targeted degree (*p* > 0.05). For sedentary behavior, significant differences were found for field of study (*F*_(5, 3816)_ = 5.69, *p* < 0.001, ηp2 = 0.01) and targeted degree (*F*_(3, 3868)_ = 3.94, *p* < 0.01, ηp2 = 0.003), but not for gender, age and study semester (*p* > 0.05).

**Conclusion:**

Female students, students enrolled in “natural sciences, mathematics and informatics” and first year students appear to have an increased risk of an unhealthy lifestyle. Future research should identify barriers to and incentives of physical activity as well as reasons for high amounts of SB in sub-populations of university students. Suitable prevention and intervention programs are necessary.

## Introduction

A conscious lifestyle can contribute to the long-term maintenance of health and wellbeing at any age. In this context, regular physical activity (PA) is a key factor preventing non-communicable diseases and primary causes of premature morbidity and mortality. Nevertheless, about 31 % of all adults worldwide are physically inactive, meaning they do not meet the minimum recommendation of PA consolidated by professional health societies like the World Health Organization (WHO) (1). The WHO classified physical inactivity as fourth leading risk factor for global mortality (2), not only negatively affecting the individual, but also representing a significant economic burden (3). To counteract the negative effects of physical inactivity and promote health, the WHO recommends at least 150 min of at least moderate or 75 min of vigorous physical activity per week, complemented by strength training twice a week (4–6). Regarding the total PA, which includes light-intensity PA like walking, the highest health-gains are reported to be occurring at 3,000 metabolic equivalent of task- (MET-) min/week (2, 7).

Regardless of the PA performed, sedentary behavior (SB) is another factor strongly influencing health and wellbeing. Tremblay (8) defines SB as activities with an energy expenditure below 1.5 MET, such as lying or sitting still, whereas physical inactivity means an insufficient amount of moderate to vigorous PA (MVPA). SB has become more and more prevalent in modern societies due to changes in the physical, social, and economic environments. Independent of but equal to PA, total sitting time is associated with a greater risk for several major chronic diseases and all-cause mortality (9–12). For an increased risk for all-cause and cardiovascular mortality, a threshold of 6 to 8 h/day of total sitting was identified (13, 14). A recent review reports that adults worldwide spend an average of 6.4 h per day sitting, ranging from 3.8 to 11.9 h (15). In comparison to that, objective measurement methods revealed even higher sitting times (median 8.2 h/day) than self-reported assessments (5.5 h/day) (15). A meta-analysis from Ekelund et al. (16) observed that about 1 h of MVPA per day is necessary to mitigate an increased all-cause mortality risk due to such high sitting time. Therefore, public health strategies have to focus on enhancing PA and prolonged SB simultaneously (17, 18).

The transition from school to university is a time that leads to changes in the home environment, work environment and leisure time. This change in living environment is often described as critical phase potentially vulnerable to risk behaviors, such as alcohol consumption and lack of physical activity (19, 20). Already as high school progresses, a significant decline of pupils meeting the minimum age-appropriate (5–17 years) recommendations for PA (60 min of MVPA per day) is evident (21). The situation is similar for university students, where only about 50 % achieved the recommendations for PA (22–25). Additionally, students' everyday life is characterized by sedentary activities (e.g., visiting lectures, classes and seminars, studying) (26–28). Therefore, it is not surprising, that the prevalence of SB appears to be much higher in university students than the global average (29). Regarding total SB, self-reported estimates across 32 studies indicate that university students spend on average 7.29 h per day sitting (30). During study semester, previous research found an increase of levels of PA (31, 32). In addition, SB is expected to increase as study progresses (30), which is consistent with the increase of weight and body fat percentage (33). However, health promotion in universities offers not only the opportunity to positively influence students' health behavior, but is also beneficial for general society since students are the leaders, decision-makers, and parents of tomorrow (34).

In order to promote health, it is necessary to identify potential health-related risk groups within the student population. In this context, the field of physical health is understudied (35). Moreover, the influence of study-related factors like study semester and major field of study on student health was either not investigated or yielded inconsistent results (30–32, 35).

Therefore, the aim of this study was (1) to assess the amount of PA and SB in German university students and (2) to identify subgroups in this population with increased risk for poor health. To identify potential health-related risk groups, differences in gender and age as well as study-related factors such as field of study, targeted degree and study semester were investigated.

## Materials and Methods

### Study Design and Ethical Approval

In summer term 2019, a cross-sectional online survey was conducted as part of an ongoing evidence-based student health initiative at the Johannes Gutenberg-University of Mainz (JGU, Germany). It included around 270 items regarding important health-related factors from mostly validated standard instruments and partly self-constructed or adapted items. The web-based software Unipark (Tivian XI, Cologne, Germany) was used to design the questionnaire. Pre-tests were conducted to examine question presentation, completion time and question comprehension, resulting in minor adaptions of the questionnaire (36). The survey was online for 49 days and the students received 4 reminders during this period. The survey was approved by the local ethics committee of the Medical Association of Rhineland-Palatinate (application-number: 2019-14336). All participants provided digital informed consent. More in-depth information regarding the survey methodology and the questionnaire is provided in the publication of Reichel et al. (36).

### Participants

All students being enrolled in the summer term of 2019 in at least one subject at the JGU (*N* = 31,213) were invited to take part in the survey. The JGU is organized in ten faculties and additionally the Mainz School of Music and the Mainz Academy of Fine Arts. Study subjects range from law and economy over social- and natural sciences, humanities and medicine to music, fine arts and sport. All students received a link to the survey via the university mailing list. Monetary and non-monetary incentives were held out to increase motivation to participate.

### Measures

The German short form of the International Physical Activity Questionnaire (IPAQ-SF) was used to assess the self-reported PA level and sitting time (37). The IPAQ is a reliable and valid tool (38, 39) and is suitable as well as recommended to assess the PA level among university students (40, 41). The questionnaire consists of seven questions, assessing the frequency (in number of days) and duration (minutes per day) spent for (1) vigorous-intensity activities, (2) moderate-intensity activities and (3) walking over the last seven days (42). Additionally, the time spent sitting on a weekday was assessed as an indicator for SB (42). The sums of (1) and (2) were cumulated to calculate the amount of MVPA in minutes per week.

In addition to the IPAQ-SF, the self-reported sociodemographic variables gender, age, as well as study related variables such as field of study, targeted degree and study semester were assessed to identify sub-groups for an inactive and sedentary lifestyle among university students.

### Data Processing and Statistical Analyses

The predefined protocol from Cheng (43) was used to calculate the overall PA expressed in MET-minutes/week. According to the guidelines for data processing and analysis of the IPAQ-SF, questionnaires were considered invalid, if any variable was missing, or the total sum of walking, moderate and vigorous activity as well as the total sum of time spent sitting per day exceeded 960 min (16 h) (44). The collected data can be summarized as a continuous indicator for PA expressed in MET-minutes/week, commonly used to assess total PA (45). Therefore, the weekly time for moderate and vigorous activity as well as walking were computed by separately multiplying the minutes per day and the days per week. The calculated minutes per week for each category were multiplied by MET (expressed as MET-minutes per week) to weight each type of activity by its energy expenditure. Time spent in low-intensity activities, such as walking, is multiplied by 3.3, time spent in moderate-intensity activities are multiplied by 4, and time spent in high-intensity activities are multiplied by 8 (44).

The data on MVPA and vigorous intensity PA were used to ensure the fulfillment of the PA-recommendations of professional health associations and therefore classified as insufficiently, moderately or highly active (4, 46). On that account, individuals not meeting the minimal suggestions of at least 150 min of MVPA or 75 min of vigorous PA are classified as insufficiently active. Participants meeting the suggestions for additional health benefits of at least 300 min of MVPA or 150 min of vigorous PA are classified as highly active, while those only achieving the minimum requirements of PA being categorized as moderately active.

The reported time spent sitting per weekday in the last seven days is presented in minutes per day according to the predefined scoring protocol (44). Referring to current scientific findings of sitting 8 h per day being associated with significantly increased risk of mortality (13, 14, 16, 47), time spent sitting was dichotomized into sitting <8 h and sitting at least 8 h.

Participants were dichotomized by the median age value into those, who are maximal 23 years old and those being older. Based on BMI, all students were classified as underweight (BMI <18.5), normal weight (18.5 ≤ BMI ≤ 24.9), pre-obesity (25.0 ≤ BMI ≤ 29.9) and obesity (BMI ≥ 30) (48).

In accordance with previous studies (31, 49, 50) and the organization of the university in different faculties, students' field of study was allocated to the following groups: “natural sciences, mathematic and informatics,” “social sciences, media and sport,” “language, humanities and cultural studies,” “medicine,” “economics and law” and “education.”

Statistical analyses were performed using SPSS version 23 (IBM, Chicago, IL, USA). Descriptive analysis of overall PA and the proportions of insufficiently, moderately and highly active participants as well as sitting time and proportions of sitting times of at least 8 h were computed for all participants, and separately for sociodemographic and study-related variables. To identify subgroups with increased risk for an unhealthy lifestyle, differences between gender, age groups, targeted degree, field of study and study semester were performed for mean values of PA (MET-minutes/week) and SB (minutes/day sitting). Homogeneity of variances was assessed using Levene‘s Test. If equal variances could be assumed, a single factorial ANOVA with Tukey correction was performed, otherwise a Welch-ANOVA with Games-Howell correction was carried out. The effect size was estimated by partial eta^2^ (ηp2) with ηp2 ≥ 0.01 indicating a small, ηp2 ≥ 0.06 a medium, and ηp2 ≥ 0.14 a large effect (51). Statistical significance was set at probability values < 0.05 (*p* < 0.05).

## Results

### Sample Characteristics

A total of 5,006 participants viewed the first page of the questionnaire. 4,714 students continued further. After a manual data cleaning according to predefined criteria, the final sample was 4,351, demonstrating a response rate of 13.9 % of the whole student body (36). After processing data on PA acording to the guidelines for data processing and analysis of the IPAQ-SF (44), 3,961 participants were included in this study. Demographic data on gender, age, and BMI, as well as targeted degree and study semester of all included students were shown in Table 1. The distribution of students on the field of study, overall and stratified by gender and study semester were shown in Table 2.

**Table 1:** Sample characteristics relating to gender, age, BMI, targeted degree and study semester.

**Characteristics**	**Value**
Gender*, N* (%)	3,961 (100)
Female *N* (%)	2,830 (71.4)
Male *N* (%)	1,100 (27.8)
Diverse *N* (%)	31 (0.8)
Age, *N* (%)	3,958 (99.9)
range, years (mean ± SD; Median)	16–73 (23.8 ± 4.3; 23.0)
BMI (mean ± SD), *N* = 3,928 (99.2%)	23.1 ± 4.2
Underweight, *N* (%)	235 (5.9)
Normal weight, *N* (%)	2,780 (70.2)
Pre-Obesity, *N* (%)	684 (17.3)
Overweight, *N* (%)	229 (5.8)
Targeted degree, *N* (%)	3,961 (100)
Bachelor	2,074 (52.4)
Master	842 (21.3)
State examination	869 (21.9)
Ph.D.	138 (3.5)
Study semester, *N* (%)	3,857 (97.4)
First year students, *N* (%)	639 (16.1)

**Table 2:** Sample characteristics relating to field of study, overall and stratified by gender and study semester.

**Field of study**	***N* (%)**	**Male *N* (%)**	**Female *N* (%)**	**Diverse *N* (%)**	**First year *N* (%)**	**Higher year *N* (%)**
Natural sciences, mathematics and informatics	712 (18.0)	306 (43.0)	403 (56.6)	3 (0.4)	117 (16.9)	575 (83.1)
Social sciences, media and sport	720 (18.2)	156 (21.7)	560 (77.8)	4 (0.6)	99 (14.3)	594 (85.7)
Language, humanities and cultural studies	795 (20.1)	143 (18.0)	632 (79.5)	20 (2.5)	115 (14.7)	665 (85.3)
Medicine	527 (13.3)	143 (27.1)	384 (72.9)	0 (0.0)	76 (14.6)	445 (85.4)
Economics and law	508 (12.8)	155 (30.5)	352 (69.3)	1 (0.2)	119 (24.2)	373 (75.8)
Education	612 (15.5)	160 (26.1)	450 (73.5)	2 (0.3)	98 (16.5)	497 (83.5)

### Physical Activity

Overall, 22.4 % of the sample were assigned as insufficiently active. Stratified by gender, 17.6 % of male, 24.2 % of female and 32.3 % of diverse students were insufficiently active. The median value of PA was 3,066 MET-minutes/week, with a first quartile at 1,704 MET-minutes/week. Table 3 summarizes the descriptive data as well as the results of the ANOVA or Welch's Test. There were no significant differences between age (*F*_(1, 3957)_ = 0.51, *p* > 0.05, ηp2 = 0.000) and targeted degree (*F*_(3, 609.91)_ = 1.13, *p* > 0.05, ηp2 = 0.001).

**Table 3:** Means, standard deviations, and analysis of variance in PA.

	**PA (MET-minutes/week)**			**insufficiently active**	**moderately active**	**Highly active**
	***N***	**Mean**	**SD**	***N* (%)**	***N* (%)**	***N* (%)**
Total	3,961	3,798	2,859	889 (22.4)	696 (17.6)	2,376 (60.0)
**Gender**						
Female	2,830	3,636	2,792	685 (24.2)	546 (19.3)	1,599 (56.5)
Male	1,100	4,237	2,993	194 (17.6)	145 (13.2)	761 (69.2)
Diverse	31	3,068	2,401	10 (32.3)	5 (16.1)	16 (51.6)
	**F (df)**	***p***	${\text{η}}_{\text{p}}^{\text{2}}$			
**Between-subject factor**	17.79 (2, 80.46)	<0.001	0.009			
**Age**						
≤ 23 years	2224	3770	2841	505 (22.7)	387 (17.4)	1332 (59.9)
>23 years	1734	3836	2883	384 (22.1)	309 (17.8)	1041 (60.0)
	**F (df)**	***p***	${\text{η}}_{\text{p}}^{\text{2}}$			
**Between-subject factor**	0.51 (1, 3,957)	>0.05	0.000			
**Targeted degree**						
Bachelor	2,074	3,769	2,939	505 (24.3)	385 (18.6)	1,184 (57.1)
Master	842	3,820	2,928	181 (21.5)	146 (17.3)	515 (61.2)
State examination	869	3,908	2,610	161 (18.5)	130 (15.0)	578 (66.5)
Ph.D.	138	3,518	2,578	30 (21.7)	28 (20.3)	80 (58.0)
	**F (df)**	***p***	${\text{η}}_{\text{p}}^{\text{2}}$			
**Between-subject factor**	1.13 (3, 609.91)	>0.05	0.001			
**Field of study**						
Natural sciences, mathematics and informatics	712	3,428	2,673	202 (28.4)	114 (16.0)	396 (55.6)
Social sciences, media and sports	720	3,844	2,820	134 (18.6)	145 (20.1)	441 (61.3)
Language, humanities and cultural studies	795	3,553	2,842	222 (27.9)	154 (19.4)	419 (52.7)
Medicine	527	3,981	2,574	83 (15.7)	84 (15.9)	360 (68.3)
Economics and law	508	3,801	2,726	104 (20.5)	79 (15.6)	325 (64.0)
Education	612	4,312	3,278	127 (20.8)	104 (17.0)	381 (62.3)
	**F (df)**	***p***	${\text{η}}_{\text{p}}^{\text{2}}$			
**Between-subject factor**	7.41 (5, 1738.09)	<0.001	0.01			
**Study semester**						
First year students	639	3,574	2,712	150 (23.5)	120 (18.8)	369 (57.7)
Higher year students	3,218	3,853	2,888	708 (22.0)	557 (17.3)	1953 (60.7)
	**F (df)**	***p***	${\text{η}}_{\text{p}}^{\text{2}}$			
**Between-subject factor**	5.53 (1, 948.12)	<0.05	0.001			

*Differences in gender, age, targeted degree, field of study and study semester. Prevalence of insufficiently, moderately and highly active students stratified by gender, age, targeted degree, field of study and study semester*.

With regard to gender differences, male students reported the highest and diverse students the lowest average PA values. The mean level of PA differs statistically significant for gender with small effect size (*F*_(2, 80.46)_ = 17.79, *p* < 0.001, ηp2 = 0.009). *Post-hoc* analysis revealed a significant difference between male and female students (*p* < 0.001) and between male and diverse students (*p* < 0.05), but not between female and diverse students (*p* > 0.05).

With regard to differences concerning field of study, students of natural “sciences, mathematics and informatics” (3,428 MET-min/week) and those of “languages, humanities and cultural studies” (3,553 MET-min/week) reported the lowest total PA. Students of social sciences, media and sports (3,844 MET-min/week), of medicine (3,981 MET-min/week) and those of education (4,312 MET-min/week) reported the highest total PA (Table 3). *Post-hoc* analyses revealed a significant difference between students enrolled in “natural sciences, mathematic and informatics” and those of “social sciences, media and sports” (*p* < 0.05), those of “medicine” (*p* < 0.01) and those of “education” (*p* < 0.001). In addition, a significant difference was found between students of “languages, humanities and cultural studies” and students of “education” (*p* < 0.001). Common to all fields of study, female students reported lower PA values than male students. The gender difference on PA was highest among those studying in the field of “education” (-1,107 MET-min/week) and lowest for students of “natural sciences, mathematic and informatics” (-144 MET-min/week).

With regard to study semester, first year students reported significant lower levels of PA than students of higher years with negligible effect (*F*_(1, 948.12)_ = 5.53, *p* < 0.05, ηp2 = 0.001) (Table 3).

### Sedentary Behavior

The average self-reported sitting time of university students is 7h 25min, with 47.6 % of the students sitting at least 8h per weekday. Table 4 summarizes the descriptive data as well as the results of the ANOVA or Welch's Test. There were no significant differences between age (*F*_(1, 3608.01)_ = 2.10, *p* > 0.05, ηp2 = 0.001), gender (*F*_(2, 74.80)_ = 0.48, *p* > 0.05, ηp2 = 0.000) and study semester (*F*_(1, 3802)_ = 0.49, *p* > 0.05, ηp2 = 0.000).

**Table 4:** Means, standard deviations, and analysis of variance in SB.

	**Sitting time (minutes/day)**			**Sitting time <8 h**	**Sitting time ≥8h**
	***N***	**Mean**	**SD**	***N* (%)**	***N* (%)**
Total	3,906	445	164	2,047 (52.4)	1,859 (47.6)
**Gender**					
Female	2,783	446	161	1,443 (51.9)	1,340 (48.1)
Male	1,094	442	173	593 (54.2)	501 (45.8)
Diverse	29	466	153	11 (37.9)	18 (62.1)
	**F (df)**	***p***	${\text{η}}_{\text{p}}^{\text{2}}$		
**Between-subject factor**	0.48 (2, 74.80)	>0.05	0.000		
**Age**					
≤ 23 years	2,193	442	162	1,187 (54.1)	1,006 (45.9)
>23 years	1,709	449	167	857 (50.1)	852 (49.9)
	**F (df)**	***p***	${\text{η}}_{\text{p}}^{\text{2}}$		
**Between-subject factor**	2.10 (1, 3608.01)	>0.05	0.001		
**Targeted degree**					
Bachelor	2,034	438	167	1,124 (55.3)	910 (44.7)
Master	832	447	162	420 (50.5)	412 (49.5)
State examination	866	454	160	423 (48.8)	443 (51.2)
Ph.D.	137	476	159	62 (45.3)	75 (54.7)
	**F (df)**	***p***	${\text{η}}_{\text{p}}^{\text{2}}$		
**Between-subject factor**	3.94 (3, 3,868)	<0.01	0.003		
**Field of study**					
Natural sciences, mathematics and informatics	707	467	166	333 (47.1)	374 (52.9)
Social sciences, media and sports	713	427	163	419 (58.8)	294 (41.2)
Language, humanities and cultural studies	773	450	167	410 (53.0)	363 (47.0)
Medicine	524	442	159	268 (51.1)	256 (48.9)
Economics and law	507	455	157	243 (47.9)	264 (52.1)
Education	593	430	166	324 (54.6)	269 (45.4)
	**F (df)**	***p***	${\text{η}}_{\text{p}}^{\text{2}}$		
**Between-subject factor**	5.69 (5, 3,816)	<0.001	0.007		
**Study semester**					
First year students	636	441	166	343 (53.9)	293 (46.1)
Higher year students	3,167	446	163	1,655 (52.3)	1,512 (47.7)
	**F (df)**	***p***	${\text{η}}_{\text{p}}^{\text{2}}$		
**Between-subject factor**	0.49 (1, 3,802)	>0.05	0.000		

*Differences in gender, age, targeted degree, field of study and study semester. Prevalence of sitting time (<8 vs. ≥8 h) stratified by gender, age, targeted degree, field of study and study semester*.

The self-reported time spent sitting differs significantly between the groups assigned according to the targeted degree with negligible effect size (*F*_(3, 3868)_ = 3.94, *p* < 0.01, ηp2 = 0.003). *Post-hoc* analysis revealed students targeting a bachelor's degree differed statistically significant from those targeting a Ph.D. (*p* < 0.05). Students targeting a Ph.D. reported the highest average sitting time of 7 h 56 min and highest prevalence (54.7 %) of sitting at least 8 h per weekday.

With regard to field of study, differences in the self-reported time spent sitting differed significantly with negligible effect size (*F*_(5, 3816)_ = 5.69, *p* < 0.001, ηp2 = 0.007). *Post-hoc* analysis revealed that students of “social sciences, media and sports” differed statistically significant from those of “natural sciences, mathematic and informatics” (*p* < 0.001) and from those of “economics and law' (*p* < 0.05). Additionally, a statistically significant difference was found between students of “natural sciences, mathematic and informatics” and those of “education” (*p* < 0.001). The highest average sitting time of 7 h 47 min was reported by students of “natural sciences, mathematic and informatics,” which are also showing the highest prevalence (52.9 %) of sitting at least 8 h per day. Students of “social sciences, media and sports” stated the lowest average sitting time of 7 h 8 min and lowest prevalence rates of sitting at least 8 h per day (41.2 %) compared to students of other fields of study.

## Discussion

In the context of a university-based health promotion program, we investigated PA and SB of students enrolled in the University of Mainz. About 22.4 % of all students that participated in this study did not reach the WHO recommendations for physical activity. In addition, 47.6 % of students sat for 8 h or more a day. This magnitude of physical inactivity and SB negatively affects health and contributes to the development of diseases (18). We found significant differences between female and male students regarding PA but not regarding SB. Furthermore, we revealed significant differences between the fields of study for both PA and SB. Especially students in the field of “natural sciences, mathematic and informatics” showed increased amounts of physical inactivity and high levels of SB. In comparison, the students in the fields of “medicine” and “education” showed high activity rates. The field of “social sciences, media and sports” was related to lower SB. Students targeting a Ph.D reported significant higher sitting times compared to those targeting a bachelor. There were no significant group-differences for students age and the study semester.

### Physical Activity

Recent studies of PA-engagement of adults in Germany revealed a prevalence of at least 47.2 %, who do not meet the PA recommendations (45, 52), which is higher, than the average high-income Western countries (36.8 %) and the global average (27.5 %) (29). The higher rates of university students fulfilling PA recommendations was expected due to the typically younger age of students of 23.8 ± 4.3 years compared to the general population (45, 52). Previous research on 18 to 29 year old persons in Germany found a proportion of 43 % not meeting common PA recommendations (45, 52) indicating that university students are not only an active population (53), but more active than the age-matched peers in the normal population. International studies on self-reported PA in university students show inconsistent results with inactivity rates ranging from 22 to 79.8 % (22–25, 54–57). Recent investigations on PA in German university students estimated, that approximately 43.6 to 53.9 % of the participants (50, 54) do not meet the PA recommendations, and show, thus, a lower prevalence than this study. However, the surveyed data are difficult to compare to the present study due to different measuring instruments. Altogether, the results of inactivity rates in university students of the present study align directly with previous research when comparable measurement tools were used.

To further increase physical activity in German university students, barriers and motivators of physical activity should be considered when implementing health interventions. The reasons for and against physical activity among university students are very diverse (55). Lack of time, bad weather, and discomfort were highlighted as barriers, whereas health consciousness, weight loss, and stress management were mentioned as motivators (55). Risk groups might help to specify the barriers and incitements of university students.

In this context, our results showed men being significantly more active than women, which aligns with previous studies of the global (56), European (53), and German adult population (52, 57), as well as with current results from university students (31, 58–60). Nelson et al. (23) found, however, a greater extent in over-reporting of PA in men than in women, with no measurable difference between male and female university students using objective measurements. Downs et al. (59) could also show the greater extent of over-reporting PA compared to objective measurements in male students. These authors, however, objectively proved male students to be significant more physically active (45.5 % were at least moderately active) than female students (22.9 % were at least moderately active) (59). Wilson et al. (61) found an emerging discomfort in women regarding the use of recreational facilities, which could be a reason for lower engagement in physical activities. Hereby, perceived lack of skill and self-consciousness, as well as the presence of men seem to play a decisive role (61). Additionally men reported significantly higher activity rates than students assigning themselves as diverse. To date, there are no valid information on the distribution of people assigning themselves as diverse (non-binary). A percentage of 1.7 to 2.1 % of a population is assumed as diverse (62, 63). In the present study, 0.8 % (*n* = 31) assigned themselves as non-binary, which is lower than the estimated distribution, but in line with previous student health surveys in Germany (31, 50). A total of 32.3 % of the diverse students did not meet PA-recommendations. Current research in the field of physical activity and exercise does not take diverse students into account, even if the relevant data had been collected (31, 50, 60).

Several Eurobarometer Studies (53, 64) found a significant decrease of the engagement in PA from 15–24 to 25–34 year olds. In contrast to that, the results of this study cannot confirm a difference in PA between younger students (≤ 23 years) and older students (>23 years). The inconsistency in the study results could be due to the different categorization and a different age range across all students in the different studies.

To our knowledge, no previous research investigated the effect of the targeted degree on the amount of PA in university students. Although no significant difference was found, the proportion of being insufficiently active among university students targeting a Bachelor's degree (24.3 %) was higher, than the students targeting another degree (18.5–21.7 %). This may be due to the fact that the Bachelor's programme is mainly attended by people who have just started their studies or are in a low study semester. Thus, we found significant differences between first year students and those of higher years. Previous studies are in line with our results of first year students being significantly less active than those of higher years (31, 32). It has been shown, that the transition from secondary school to university is characterized by changes in lifestyle, often leading to an increased risk behavior (1).

The present results show a significant difference between fields of study and are, thus, in contrast to previous study results (31, 50, 54, 65–67). Knowledge seems to exist regarding students in the field of “medicine,” that have been reported to be more active than age-matched peers in the general population (68). Previous research is disunited in regard of PA, when compared to non-medical students (31, 67). Results of this study show medical students to be among the highly active sub-population of university students with only 15.7 % being classified as inactive. Likewise, positively accentuated seem to be students of the field of “social sciences, media and sport” as well as “education” and “economics and law.” This stands in contrast to the findings of Grützmacher et al. (31), according to which students in the field of social sciences and education were less physically active compared to other fields of study. A possible explanation for the different results compared to other studies could be an inconsistent categorization of the fields of study. The present survey tried to establish a possible universally applicable division of the fields of study based on the pre-work of Dietz et al. (49) and Grützmacher et al. (31).

### Sedentary Behavior

In regard of SB, the overall self-reported sitting time in the present study was 445 min (7.42 h). This is 8 min higher (7.29 h) than the average university student, as a recent meta-analysis on SB of university students stated (30). Thereby, Castro et al. (30) reviewed studies being conducted globally, with 32 % carried out at European universities. The average adult (>18 years) in Europe, as well as in Germany, usually sits for 5 h per day (69, 70). Age peers in Europe were also sitting for 5 h (18–24 years old), or 4 h per day (25–34 years old), respectively (70). The peers in Germany (18–29 years old) were sitting on average 6.17 h per day (69). Therefore, the presented data demonstrate, that students of the university of Mainz usually sit longer compared to age- and regional peers and compared to previous studies on university students (30). The high amount of sedentary behavior in university students is attributed to the typical activities associated with studying (26–28, 30). These are comparable to those of desk workers sitting on average 7 h per day (70). A threshold of 6 to 8 h of self-reported sitting time per day (14, 47), or 9.5 h of accelerometer derived sitting time per day, respectively (71), has shown to negatively affect health. Based on the results of the present study, 47.6 % of students were sitting at least 8 h per day and are, thus, exposed to an increased risk for chronic diseases. Similar to research findings in the field of PA, objectively collected data on total sitting time have shown to be even higher than self-reported data (23, 72, 73). In particular, objectively measured sitting time among university students is on average 2 h 14 min higher compared to self-reports (23), which is supported by similar results in the general population underestimating SB by 2.2 to 4 h per day (72, 73).

Prior research reported significant gender differences in sitting time of German adults in favor of men (69). However, there were no gender differences found in the age group of 18 to 29 year olds (69) and in university students (30), which aligns with the results of the present study. To date no valid information on SB regarding non-binary people exists. It might be that the factors explaining variation in university students are different from those in the general population. In addition, SB might be mostly determined by the university setting, which applies equally to all genders.

Castro et al. (30) highlights the lack of knowledge regarding the role of study related risk-factors like the field of study and the targeted degree on SB in university students. The present study found statistically significant differences in the reported sitting time in relation to the targeted degree. Hereby nearly 55% of Ph.D. students reported sitting times of at least 8 h per day with an average sitting time of 7 h 56 min per day, whereas bachelor students reported the lowest average sitting time and highest prevalence of sitting at least 8 h per day. Regarding the targeted degree, SB seems to increase with increasing academic skill level.

We found significant differences in sitting time between the fields of study. Lower sitting times were found in students in the fields of “social sciences, media and sport” still reporting 7 h 7 min of daily sitting time. With a prevalence of 52.9 %, students in the fields of “natural sciences, mathematic and informatics” sit at least 8 h per day. This highlights this sub-group of being at increased risk for negative health effects due to the high amounts of SB.

The study semester had no effect on increasing or decreasing daily sitting time. This is in contrast to current research expecting higher workload with increasing semester and, thus, accumulating more sitting hours in higher year students (30). On the other hand, a reason for higher sitting times in first year students could be longer studying phases due to the lack of individual learning strategies compared to higher year students who may have already developed suitable learning strategies.

### Associations of Physical Activity and Sedentary Behavior

Physical inactivity and sedentary behavior are independent risk factors for all-cause mortality, cardiovascular disease mortality and non-communicable diseases (NCD) among others (46). Nevertheless, Castro et al. (74) reported a negative association between physical inactivity and SB in university students (74). A small to medium negative association between MVPA and SB and medium to large association between light-intensity PA and SB was also found in adults (75). Regarding our results, it needs to be highlighted, that students of “natural sciences, mathematic and informatics” are significantly less active and significantly more sedentary. Hereby, especially in the field of “language, humanities and cultural studies” the proportion of female students is higher than the average. Female students have shown to be less active and more sedentary than male students.

### Limitations and Future Research

First of all, a possible selection bias of health interested students should be acknowledged and might, therefore, have positively influenced the outcomes for PA and SB (45). Due to the overall length of the questionnaire, it was not possible to differ between weekdays and weekend-days or assess domain-specific distinction concerning PA and SB. Some students may pay attention to a healthy lifestyle, especially on weekends, when they do not have to attend to lectures and seminars. This should be considered in future studies. In addition, the reported outcomes are based on self-reports of PA and SB and could be biased by false information due to social desirability (69). Although the IPAQ-SF is a reliable and valid self-report measurement to assess PA (38, 39, 76) and is recommended to assess the PA level of university students (40, 41), scientific investigations comparing self-reported and objectively measured PA in undergraduate students suggest an over-reporting of PA when using self-report measurements (23, 59). This strengthens the demand for further objective-obtained data in university students.

Moreover, female students account for 59 % of the university population and were overrepresented in the present study (71.4 %). Due to the small sample size of diverse students, further investigations are needed to give more insight in the PA and SB of this population. Furthermore, more research is needed to clarify the impact of the fields of study on health risk behavior in university students. Students' knowledge on health-promoting behavior seems to play an important role for a healthy lifestyle (medicine students vs. other study groups). Future studies should investigate to what extent this knowledge differs among different study subjects and in which field of study further information on health behavior should be communicated. These studies could also examine more detailed, how the program of individual fields of study is structured, how many compulsory courses there are, how many exams there are, how much time needs to be spent studying. In this course it seems important to investigate to what extent learning phases before exams affect the amount of PA and SB in university students. Furthermore, it could be investigated which sporting activities are offered at the university and in the city of Mainz, how the numbers of participants and age as well as gender distributions are. This information might help to subsequently recommend suitable sports or sports groups to the corresponding risk groups or to create more suitable offers.

Due to the small effect sizes, further investigations are necessary to verify our results and confirm the risk groups we have identified.

### Practical Relevance–Promoting a Healthy Lifestyle

The results from the present investigation are valuable as they help to identify segments of the student population which may be at greater risk for engaging in less PA and higher volumes of SB. In turn, this information can identify target audiences for policies and interventions on reducing SB and promoting PA in university settings. Although interventions to implement and evaluate a healthy lifestyle seem to be effective in tertiary institutions (77), to date interventions on improving PA and reducing SB in university students remain rare. In this regard, especially individual compared to group interventions showed good effects on health risk (35). However, according to the setting approach, interventions should include larger groups with risky health behavior.

On that account, strategies on reducing SB and promoting physical activity are complementary approaches with individual focus and implementations, representing a dual strategy (30, 78). The significant lower self-reported PA of female students and of non-binary students of the present survey highlight the demand for further gender-sensitive investigations in the field of health behavior as well as interventions adapted to the needs of these groups. Moreover, educational advertising and specific interventions are worthwhile to already take place at the beginning of studies. In addition, a special focus should be placed on the specific characteristics of the fields of study, like “natural sciences, mathematic and informatics” and regarding the targeted degree Ph.D. when applying for health promotion.

Maselli et al. (1) conclude, that effective interventions to promote PA in university students should focus on behavioral determinants. Among others, interventions using internet-based, stage-matched messages (79), Tai Chi (80) and social cognitive PA interventions (81) were found to benefit health in university students. Regarding SB, breaking up prolonged sitting with frequent bouts of standing or light-intensity PA have shown to improve stroke risk factors (82). Interventions aiming to reduce negative effects due to SB should therefore, not exclusively focus on reducing overall time being sedentary, but also breaking up prolonged sitting with bouts of light-intensity PA or standing (83). Future investigations should focus on the effectiveness of interventions targeting specific risk groups of health among the student population.

## Conclusion

In summary, our study results showed a high level of PA combined with high amounts of SB in university students. Consequently, this population requires specific interventions that particularly counteract high sitting time. To identify subgroups of increased risk for a poor lifestyle, we examined sociodemographic and study-related differences in PA and SB behaviors. Female students, students from the field of “natural sciences, mathematic and informatics,” Ph.D.-students and first year students, seem to be subgroups at increased risk. Based on these findings, prevention and intervention models need to be established in university health-promotion programmes, in which the facilitation of PA and the reduction of SB in these specific subgroups should be key parts. Despite the findings in the present study, future research should evaluate a combination of objective accelerometer-derived and self-reported information as is recommended to assess PA and SB. Further, prospective research should be performed to identify possible barriers to physical activity and possible reasons for high amounts of SB in sub-populations of university students to initiate and implement suitable prevention and intervention programmes.

## Data Availability Statement

The raw data supporting the conclusions of this article will be made available by the authors, without undue reservation.

## Ethics Statement

The studies involving human participants were reviewed and approved by Ethical Committee of the Medical Association of Rhineland-Palatinate. The patients/participants provided their written informed consent to participate in this study.

## Author Contributions

Conception and design: DE, DP, PD, JR, AW, AT, MS, ND, SL, and PS. Acquisition of data: DE, DP, PD, JR, AW, AT, MS, ND, and SL. Analysis and interpretation of the data: DE, DP, SH, PS, and KK. Drafting of the article: DE, DP, and KK. Critical revision of the article for important intellectual content and final approval of the article: All authors.

## Funding

The Healthy Campus Mainz project was funded by BARMER health insurance and carried out with the support of the Johannes Gutenberg-University of Mainz and the University Medical Center of the Johannes Gutenberg-University of Mainz.

## Conflict of Interest

The authors declare that the research was conducted in the absence of any commercial or financial relationships that could be construed as a potential conflict of interest.

## Publisher's Note

All claims expressed in this article are solely those of the authors and do not necessarily represent those of their affiliated organizations, or those of the publisher, the editors and the reviewers. Any product that may be evaluated in this article, or claim that may be made by its manufacturer, is not guaranteed or endorsed by the publisher.
